# Adaptive finite difference for seismic wavefield modelling in acoustic media

**DOI:** 10.1038/srep30302

**Published:** 2016-08-05

**Authors:** Gang Yao, Di Wu, Henry Alexander Debens

**Affiliations:** 1 Department of Earth Science and Engineering, Imperial College London, London SW7 2BP, UK; 2 The Unconventional Natural Gas Institute, China University of Petroleum (Beijing), Beijing 102249, China

## Abstract

Efficient numerical seismic wavefield modelling is a key component of modern seismic imaging techniques, such as reverse-time migration and full-waveform inversion. Finite difference methods are perhaps the most widely used numerical approach for forward modelling, and here we introduce a novel scheme for implementing finite difference by introducing a time-to-space wavelet mapping. Finite difference coefficients are then computed by minimising the difference between the spatial derivatives of the mapped wavelet and the finite difference operator over all propagation angles. Since the coefficients vary adaptively with different velocities and source wavelet bandwidths, the method is capable to maximise the accuracy of the finite difference operator. Numerical examples demonstrate that this method is superior to standard finite difference methods, while comparable to Zhang’s optimised finite difference scheme.

The wave equation is a powerful mathematical tool for describing the propagation of seismic waves through the Earth. As such, seismic wavefield modelling based on the wave equation has evolved to become an essential component of advanced seismic imaging^1^,^2^,^3^,^4^,^5^ and model building techniques, such as full waveform inversion^6^,^7^,^8^. The most popular modelling method is finite difference, mainly because it is simple to implement and highly efficient compared to other techniques, such as finite element^9^. A key limitation of finite difference methods however is their susceptibility to numerical dispersion, whereby waves of different frequency possess different artificial speeds^10^. For standard finite difference based on Taylor series expansion, the higher the frequency is, the stronger the dispersion it suffers.

To mitigate this numerical dispersion, optimisation strategies are often employed to find improved finite difference coefficients (FDCs) that cover a wider frequency bandwidth and wavenumber range, but with limited errors. One such strategy is to seek optimised FDCs for spatial derivatives that are able to decrease dispersion of the spatial terms and compensate somewhat for dispersion of the temporal terms, thereby reducing the overall dispersion errors^11^,^12^,^13^,^14^,^15^. Whilst optimisations of this nature can achieve excellent compensation for one-dimensional (1D) models, the compensation deteriorates in the case of a 2D or 3D model due to the directional dependence of the apparent wavelength.

Another strategy is to find optimised FDCs that provide more accurate calculations for the spatial derivatives and therefore result in less spatial dispersion. With this strategy, the dispersion of the temporal term must be compensated for separately^16^,^17^. Windowing of the accurate differential operator in the space domain is one method for generating this kind of optimised FDCs which produce less dispersion than those of standard finite difference^18^,^19^,^20^. Optimised coefficients can also be obtained by minimising an objective function that measures the misfit between the finite difference operator and the accurate differential operator^21^,^22^,^23^.

In this paper, we present a means to calculate optimal FDCs that reduce the dispersion of the spatial terms while adaptively varying with seismic velocity and wavelet bandwidth. In this manner we can hope to exploit the maximum potential of finite difference.

## Theory

To demonstrate this method, we use the simple 2D acoustic wave equation with a source wavelet, *s*(*t*),


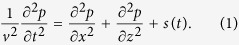


Solving equation (1) with finite difference methods entails the use of finite difference operators to numerically calculate the temporal and spatial second derivatives. Time-recursive schemes are usually employed to calculate wavefields explicitly from one time step to the next. In order to save computational memory, 2^nd^-order finite difference is commonly adopted for the temporal derivative. As a result, any improvement to accuracy and/or suppression of numerical dispersion relies on the selection of the finite difference operator for the spatial derivatives. A sensible choice is high-order finite difference, which can be written as


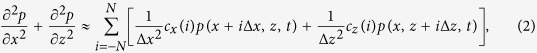


where *c*_*x*_ and *c*_*z*_ are the FDCs for the *x* and *z* derivatives, respectively, *2N* is the accuracy order of the finite difference operator, and Δ*x* and Δ*z* are horizontal and vertical sampling intervals, respectively. For waves propagating in any direction, the desired values of *c*_*x*_ and *c*_*z*_ make the right-hand side of equation (2) approximately equal to the terms of the left-hand side. Since the finite difference stencils for the *x* and *z* directions share the same form, *c*_*x*_ and *c*_*z*_ can therefore be calculated in the same way; for instance, *c*_*x*_ can be found for a waveform travelling in any direction by solving


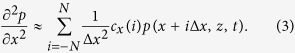


**Figure 1:**
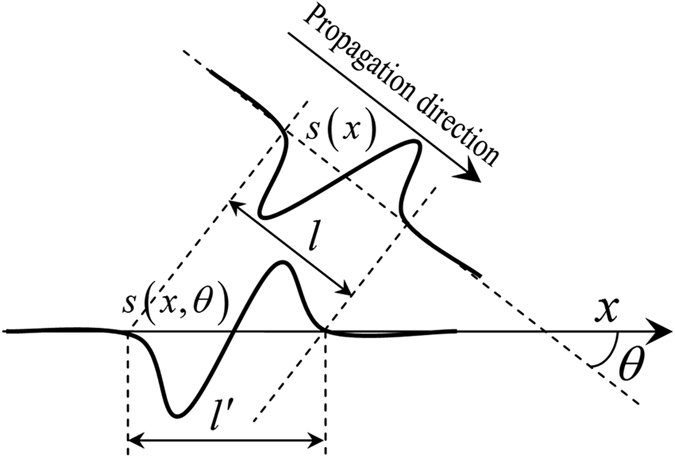
Schematic diagram of the mapping of a plane wave *s*(*x*), which has a duration of *l*, to the axis *x*. The waveform propagation direction is *θ*, defined clockwise from the axis *x*. The apparent waveform along *x* is given by *s*(*x, θ*), where its duration *l*′ is equivalent to (cos *θ*)^−1^*l*.

Since seismic wavefields are band-limited, *c*_*x*_ only needs to cover this bandwidth, which can be determined from the known source wavelet, *s*(*t*). To solve for *c*_*x*_ in equation (3), *p* can be replaced with a waveform determined by *s*. For a given velocity, the waveform of a plane wave formed by *s*(*t*) can be analytically or numerically calculated, assuming the form *s*(*x*). If considering the direction of propagation, *θ*, defined clockwise from *x* (Fig. 1), the apparent waveform of the plane wave along *x* is given by *s*(*x, θ*). The wavelength of *s*(*x, θ*) is equivalent to the wavelength of *s*(*x*) multiplied by (cos *θ*)^−1^. As a result, the second derivative, *b*(*x, θ*), of *s*(*x, θ*) can also be calculated analytically or numerically. The pseudo-spectral method^24^ gives a precise numerical solution of *b*(*x, θ*) up to the Nyquist frequency. Thus the only unknown in equation (3) is *c*_*x*_, which can be found by minimising the objective function


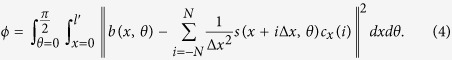


where *θ* is the wave propagation angle and *l*′ is the waveform duration. Since *b*(*x, θ*) has a linear relationship with *c*_*x*_, the minimisation of equation (4) can be effectively and efficiently achieved with gradient-based optimisation methods, for example conjugate gradient^25^.

A fixed-bandwidth wavelet will propagate with a wavelength that is dependent on the velocity of the media it is propagating through; as such the FDCs given by minimising equation (4) accordingly also change with velocity. For a heterogeneous velocity model, a lookup table of FDCs for different velocity values can be constructed prior to modelling. In practice, the coefficients only need to be calculated with a velocity interval of every few hundred metres per second. During modelling, the appropriate coefficients can then be quickly obtained as the table itself is small so can be efficiently held in cache. Wavelets with identical amplitude spectra will produce identical FDCs from equation (4). In an ideal scenario, the exact wavelet is used when computing FDCs. However, if the wavelet is unknown or difficult to determine accurately – for example during back-propagation of the adjoint source in full-waveform inversion – then we can measure the data’s frequency bandwidth and instead use a band-pass filtered spike as the wavelet in equation (4). Since the spike will have a flat frequency spectrum, the weighting for each frequency is equivalent during the minimisation of equation (4). Nonetheless, the frequency spectrum of the actual wavelet may not be flat, and as expected use of the actual wavelet produces more accurate modelling results. Numerical tests show however that this additional accuracy is relatively minor, and as such either the actual source wavelet or a band-limited spike are suitable wavelets for equation (4). Since our FDCs vary adaptively with wavelet bandwidth and velocity, this finite difference method is referred to here as adaptive finite difference (hereinafter abbreviated to AFD).

## Examples

**Figure 2:**
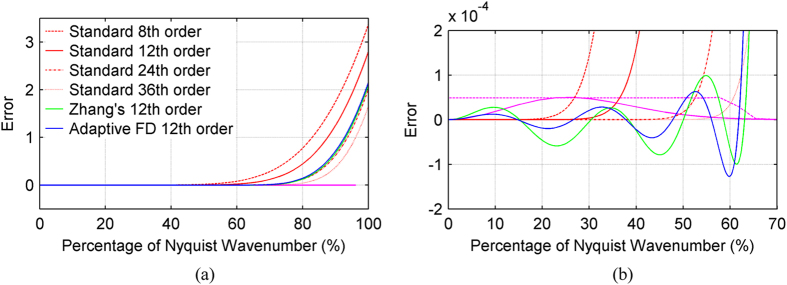
(**a**) Error as a function of wavenumber content for the SFD, Zhang’s OFD, and AFD operators, and (**b**) zoomed window of (**a**). The model has a constant velocity of 2000 m/s; the source is a 13 Hz Ricker wavelet; and the temporal and spatial sampling intervals are 0.5 ms and 20 m, respectively. The red, blue and green curves represent the SFD, 12^th^-order AFD and 12^th^-order Zhang’s OFD operators, respectively. The solid and dashed magenta curves represent the spectra of the Ricker wavelet and the band-limited wavelet used for the AFD operator calculation, respectively, both of which have been mapped into the wavenumber domain.

The first example we present here to demonstrate the performance of AFD uses a homogenous velocity model of 2000 m/s. A 13 Hz Ricker wavelet with 0.5 ms sampling interval is chosen as the source wavelet, which is located at the centre of the model. The grid size is 20 m in both the x and z directions. A band-pass filtered version of a spike with a bandwidth ranging from 0 to 32 Hz (the same frequency bandwidth as the Ricker wavelet) is chosen as the wavelet for equation (4), and hence used to calculate the FDCs. Every 4° of propagation angle – from 1° to 89° – is used when minimising equation (4). The FDCs are listed in Table 1. The errors^20^ of the 12^th^-order AFD operator, the 8^th^-, 12^th^-, 24^th^-, and 36^th^-order standard finite difference (SFD) operators, and 12^th^-order Zhang’s optimised finite difference^21^ (OFD) operator are all shown in Fig. 2. Figure 2b demonstrates that, in the region of error less than ±1.5e^−4^, AFD and Zhang’s OFD both cover a wavenumber range much broader than those of 8^th^-, 12^th^-, or 24^th^-order SFD, with both providing a good approximation to 36^th^-order SFD. This demonstrates that optimised finite difference methods can employ a much shorter operator than SFD while achieving a similar degree of accuracy. The computational cost of optimised finite difference is therefore, in turn, less than its SFD counterparts.

The AFD synthetics have smaller errors at low wavenumbers compared to those of Zhang’s OFD, but have slightly larger errors at high wavenumbers. As a result, the AFD method is more accurate for computing low-wavenumber waves. This is particularly useful for events propagating with a large angle to the *x*- and *z*-axes. The SFD operator can also achieve good accuracy at low wavenumbers, but has considerable errors at high wavenumbers. To achieve their broader wavenumber coverage, OFD operators will often act to shift the large errors seen at high wavenumbers in SFD to lower wavenumbers, with the aim of achieving minimal errors in a least-squares sense. This is the reason why OFD methods often outperform SFD methods.

**Figure 3:**
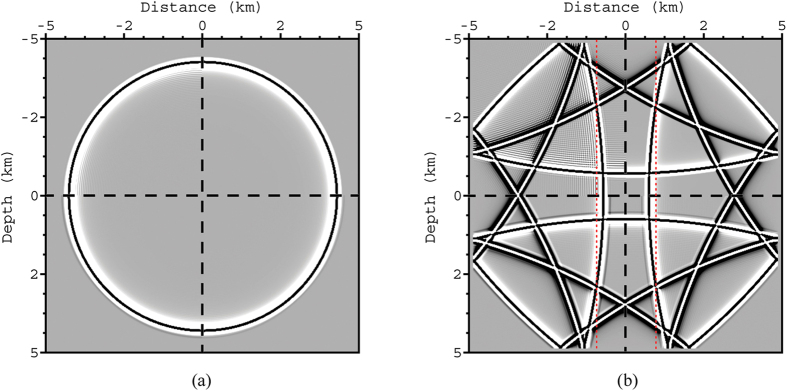
Wavefield snapshots at (**a**) 2.3 and (**b**) 9.5 s. As previously, the model has a constant velocity of 2000 m/s; the source is a 13 Hz Ricker wavelet; and the temporal and spatial sampling intervals are 0.5 ms and 20 m, respectively. The top-left quadrant is generated by the 12^th^-order SFD method; the bottom-left quadrant is given by 12^th^-order Zhang’s OFD method; the bottom-right quadrant is produced by the 12^th^-order AFD method; and the top-right quadrant is generated by the pseudo-spectral method. The displays are clipped to 3% of the maximum amplitude. In both (**a**,**b**) the SFD operator can be seen to produce more pronounced dispersion than the other three methods.

**Figure 4:**
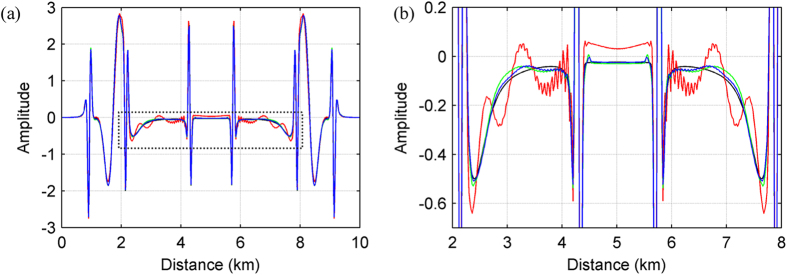
(**a**) Comparison of the waveforms in Fig. 3b along the two vertical dotted red lines and (**b**) zoomed window of (**a**) indicated by the dotted box. The black curve represents the result of the pseudo-spectral method, while the other colours match those of the methods presented in Fig. 2. As can be seen, the AFD and Zhang’s OFD methods produce waveforms with a closer match to the pseudo-spectral method than the SFD method.

**Figure 5:**
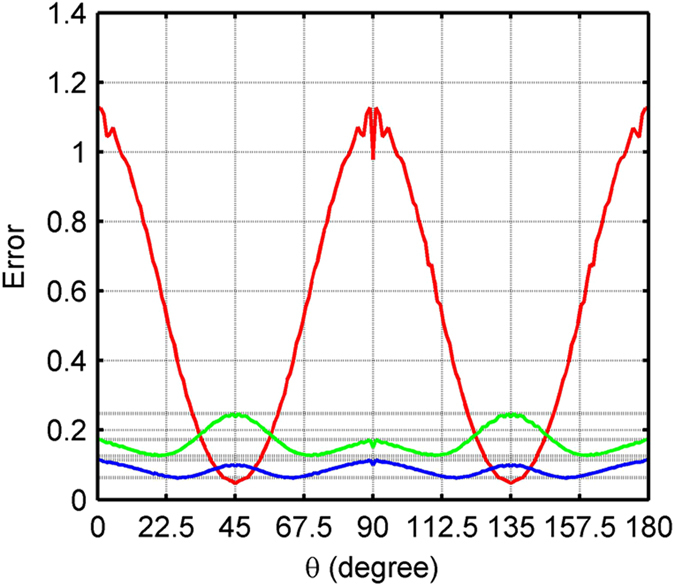
Error as a function of propagation direction for the wavefield at 2.3 s shown in Fig. 3a. The horizontal axis is propagation angle *θ*, which is defined graphically in Fig. 1. The angle has its origin at the shot position, which in this case is in the centre of the model. The vertical axis is error, determined for a single propagation angle using the ℓ^2^-norm of the wavefields produced from each finite difference method and the pseudo-spectral method. The red curve represents the 12^th^-order SFD method, the green curve 12^th^-order Zhang’s OFD method, and the blue curve the 12^th^-order AFD method. The errors of the SFD method are scaled by a factor of 0.5 for convenience.

Figure 3 shows greyscale wavefield snapshots at 2.3 and 9.5 s, while Fig. 4 shows a profile through the 9.5 s snapshot of Fig. 3b. As can be seen in these Figs, SFD incurs much stronger numerical dispersion than either AFD or Zhang’s OFD. The pseudo-spectral method achieves the least dispersion, because it calculates precise spatial derivatives up to the Nyquist wavenumber. By using the pseudo-spectral result as a reference, the AFD produces the lowest total root mean square (RMS) error, which is a measure of the degree of deviation from the reference wavefield. Furthermore, since AFD calculates FDCs by minimising equation (4) for a range of propagation angles, AFD can achieve a more balanced distribution of dispersion error across these angles. To demonstrate this, the *ℓ*^*2*^-norm (square root of the sum of the squares) error for the wavefield at 2.3 s at different angles of propagation is plotted in Fig. 5. As can be seen, the errors for AFD oscillate less than those for Zhang’s OFD. By contrast, SFD has much larger errors along the axes (0°/180° and 90°) than both AFD and Zhang’s OFD, but similar errors to AFD at intermediate orientations between the two axes (around 45° and 135°). This occurs because the apparent wavenumbers are highest along the direction of the axes, and SFD suffers from large errors at high wavenumbers (Fig. 2a).

**Figure 6:**
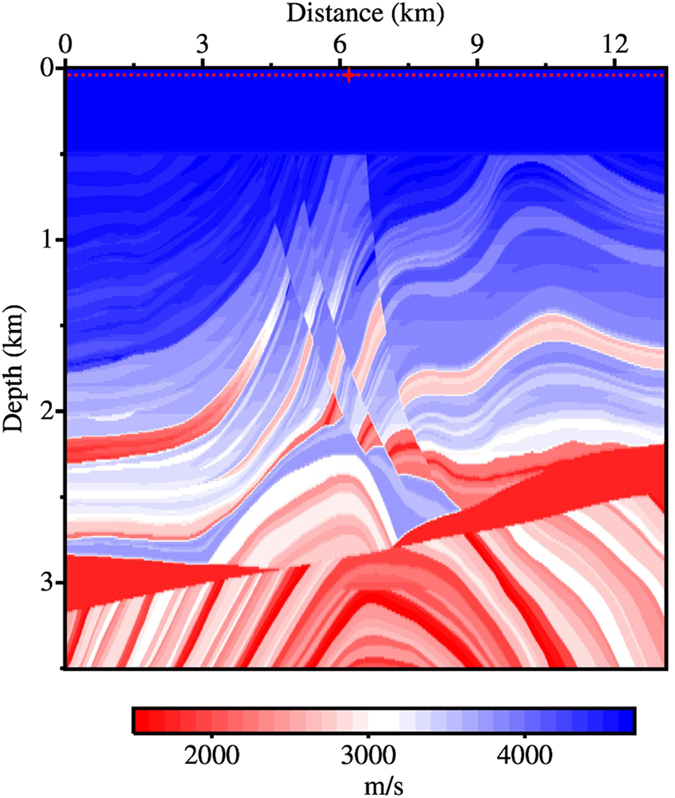
The Marmousi model. The red star indicates the location of the source, while the dotted red line represents the receiver positions.

The second example seeks to apply the same four modelling techniques to the Marmousi model (Fig. 6), discretised over a 15 m grid in both the *x* and *z* directions. A 13 Hz Ricker wavelet with 0.5 ms sampling interval is again used as the source wavelet. Different from the previous example, the Ricker wavelet, which has a frequency bandwidth of 0 to approximately 32 Hz, is used to calculate the adaptive FDCs for velocities ranging from 1500 to 4700 m/s over increments of 100 m/s. As a result, 33 sets of FDCs (Table 2) for 33 respective velocities are generated for use during modelling. Prior to modelling, an index for the FDC lookup table is pre-computed at each element of the model using the velocity of that element. Numerical tests suggest that using the velocity of each element results in less error than if taking an average velocity of neighbouring elements, even when at or in the close vicinity of velocity interfaces. The indices are stored in a single-byte array, meaning that the additional memory footprint of AFD is one quarter the size of the array that holds the model (assuming its format is single-precision floating point). The index array size is in this instance is about 288.4 kilobytes, where this extra memory cost is trivial for modern computers. By allowing the coefficients to vary with velocity in this manner, we can hope to exploit the maximum potential of the finite difference method when calculating the spatial derivatives of the wave equation.

**Figure 7:**
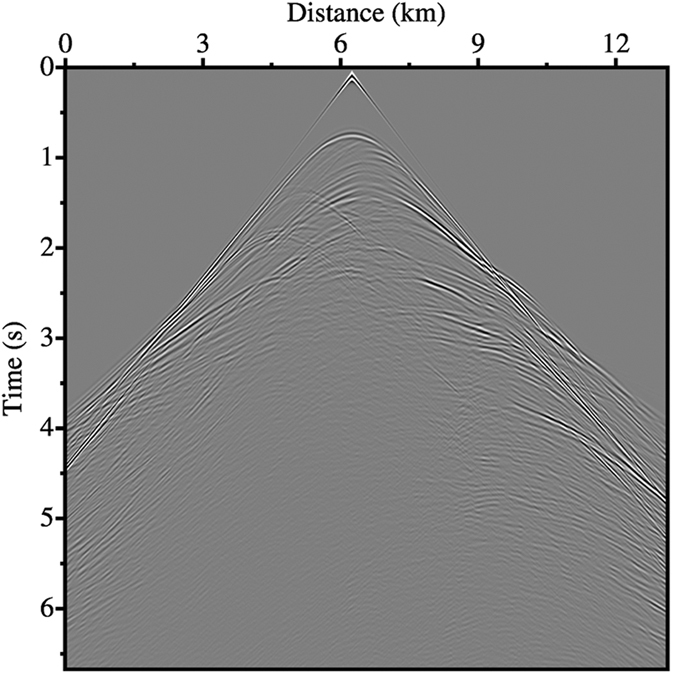
Shot record generated from the Marmousi model using the 12^th^-order AFD method. The source is located at a horizontal distance of 6.25 km and a depth of 30 m below a free surface. The receivers are positioned at the same depth as the source and distributed horizontally across the entire model.

**Figure 8:**
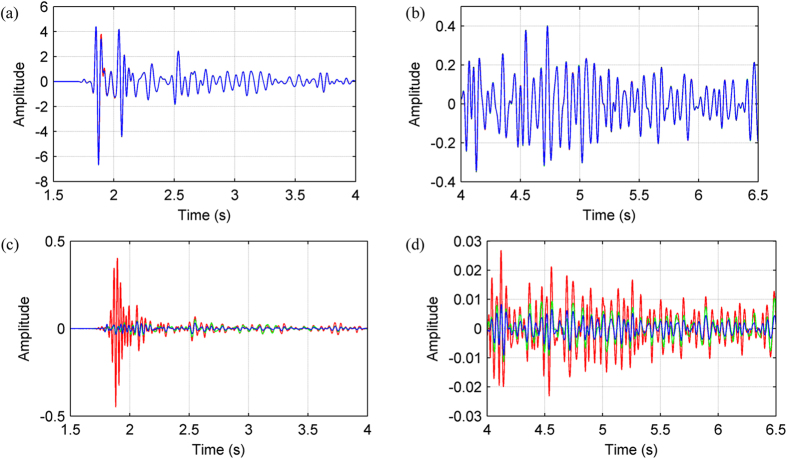
Comparison between individual traces located at a horizontal distance of 3.75 km. Panels (**a**,**b**) window the main (1.5–4.0 s) and later (4.0–6.5 s) arrivals of each synthetic seismograms, respectively. The seismograms are computed using the 12^th^-order SFD, Zhang’s OFD, and AFD methods as well as the pseudo-spectral method, and are plotted in red, green, blue, and black, respectively. Panels (**c**,**d**) contain the difference between the trace of each finite difference method and that of the pseudo-spectral method; these are plotted using the same colour scheme as in (**a**,**b**).

In order to simulate fully a marine environment, a free surface is used during modelling and the source and receivers are positioned at 30 m below the free surface. Fig. 7 shows the synthetic shot record calculated using the AFD method. At this scale, synthetic shot records generated using the other two finite difference methods appear identical to Fig. 7. In order to examine the subtleties of these synthetics, we instead choose to compare individual traces. Figure 8a,b show two time windows of the trace located at a horizontal distance of 3.75 km in each synthetic gather. For comparison, we also show the synthetic record calculated using the pseudo-spectral method. In general the differences between the four synthetic records are minimal, in both the main arrival window (Fig. 8a) and the later arrival window (Fig. 8b).

Figure 8c,d highlight the differences between the synthetic records computed using the three finite-difference methods (SFD, Zhang’s OFD and our AFD) and the pseudo-spectral method. It is now apparent that the SFD result (red) has large errors at around 1.8 s. The data in this region are dominated by shallow refracted arrivals, which have propagated through areas of low velocity. Low velocities correspond to high wavenumbers and, as demonstrated in Fig. 2b, the SFD method suffers from substantial error at large wavenumbers; consequently, the SFD result contains inaccuracies. In contrast, both the AFD and Zhang’s OFD results have much-reduced errors for these arrivals (Fig. 8c). In areas of high velocity, however, the AFD result proves more accurate than Zhang’s OFD result. This is because as the wavenumber content of the wavefield decreases in these areas, AFD modifies the FDCs such that they cover a narrower wavenumber range than those of Zhang’s OFD, which in turn results in reduced numerical dispersion. This is most noticeable for the later arrivals (Fig. 8d), which travel deeper and through regions of higher velocity.

**Figure 9:**
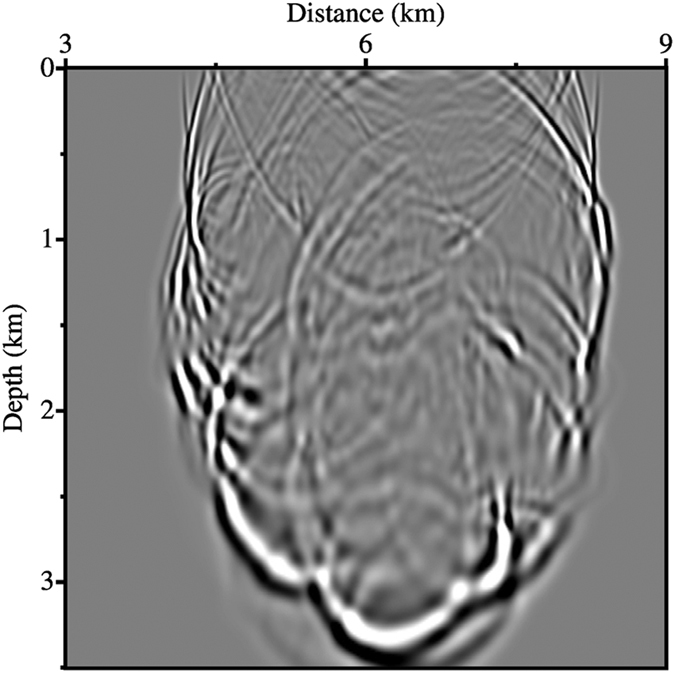
Snapshot of the AFD wavefield at 1.45 s.

**Figure 10:**
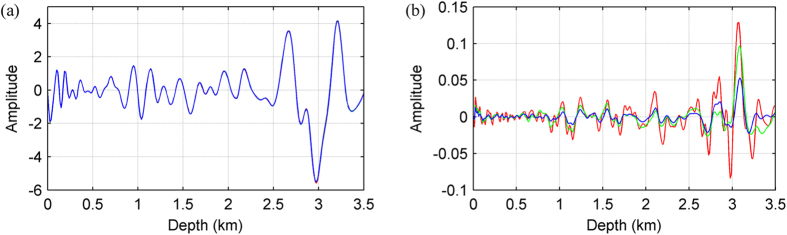
Comparison between the wavefields of Fig. 9 along a vertical profile located at a horizontal distance of 5.6 km. Panel (**a**) contains the actual wavefields, produced by the 12^th^-order SFD, Zhang’s OFD, and AFD methods along with the pseudo-spectral method. Panel (**b**) contains the difference between the record of each finite difference method and that of the pseudo-spectral method. Note that the colours used in each subfigure relate to those of Fig. 8.

Figure 9 shows a greyscale snapshot of the AFD wavefield at 1.45 s. As before, the wavefields calculated using the other two finite difference methods look very similar to this, and as such are not shown. There is noticeable difference between these synthetic wavefields when inspected in fine detail however. Figure 10 shows a comparison of the wavefields generated by the three finite difference methods and the pseudo-spectral method along a vertical profile located at a horizontal distance of 5.6 km. Figure 10b displays clearly that the AFD wavefield (blue) provides the closest match to the pseudo-spectral benchmark. Compared to that of SFD, the RMS error for AFD is 61% lower for this profile and 66% lower for the whole snapshot. Compared to the RMS error for Zhang’s OFD, the improvement provided by AFD is 42% for both this trace and the whole snapshot.

**Figure 11:**
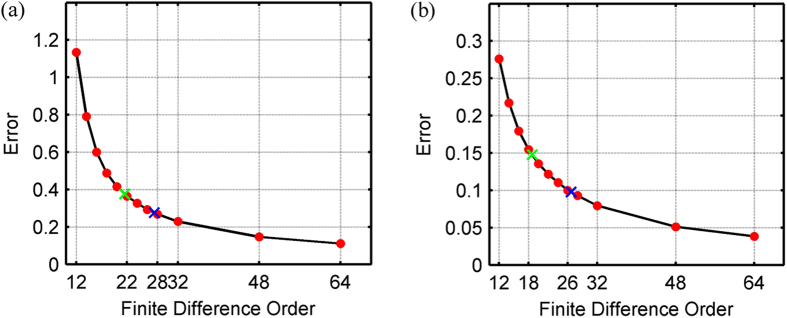
Quantification of the errors of (**a**) the shot records shown in Fig. 7 and (**b**) the wavefields at 1.45 s shown in Fig. 9. The horizontal axis is the order of finite difference, while the vertical axis is error, in this case RMS error/misfit between each finite difference result and the pseudo-spectral result. The red dots represent the RMS errors for SFD of varying order. The green and blue crosses represent 12^th^-order Zhang’s OFD and 12^th^-order AFD, respectively, where each is plotted at the corresponding order of SFD with equivalent RMS error.

We repeated the Marmousi experiment using SFD of varying order. The RMS errors for shot records (Fig. 7) produced using SFD, Zhang’s OFD, and AFD are plotted in Fig. 11a, while the RMS errors for wavefield snapshots at 1.45 s (Fig. 9) are illustrated in Fig. 11b. As can be seen in Fig. 11, the errors decrease gradually and monotonically with increasing finite difference order. Zhang’s OFD achieves almost the same accuracy as 22^nd^-order SFD for the shot record and 18^th^-order SFD for the wavefield snapshot. By contrast, AFD achieves almost the same accuracy as 28^th^-order SFD for the shot record and 26^th^-order SFD for the wavefield snapshot.

**Figure 12:**
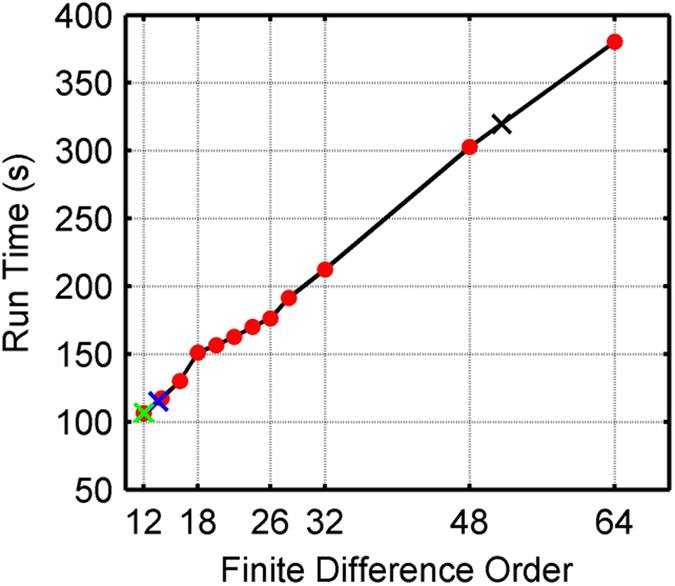
Run time of each finite difference method and the pseudo-spectral method for the Marmousi experiment. The red dots represent the run times for SFD of varying order. The green, blue and black crosses represent 12^th^-order Zhang’s OFD, 12^th^-order AFD and the pseudo-spectral method, respectively, where each is plotted at the corresponding order of SFD with equivalent run time.

Figure 12 shows the run times for this experiment. As expected, the run times of the finite difference methods increase linearly with finite difference order. The run time of AFD was in this case approximately 8% longer than that of 12^th^-order SFD and Zhang’s OFD, but shorter than that of 14^th^-order SFD. This extra computational cost associated with AFD is incurred when accessing the FDC lookup table. Interestingly, thanks to the efficiency of the fast discrete Fourier transform, the pseudo-spectral method had a shorter run time than 64^th^-order SFD in this case. However, the cost of the pseudo-spectral method is proportional to *n*·log *n*, where *n* is the linear model dimension, whereas the cost for finite difference methods is proportional to *n*. As such, use of the pseudo-spectral method often becomes computationally unappealing as the size of the model increases.

We have conducted further numerical experiments to those presented here, and just as in the examples described above, the AFD synthetics always appear to have improved accuracy over those calculated with the two other operators. This is because AFD attempts to exploit the maximum potential of finite difference by adapting the coefficients to variations in velocity.

### Discussion and conclusion

We have presented here an improved method for calculating finite difference coefficients. Since this new technique generates the coefficients in the space domain and in an adaptive manner relative to the medium velocity and bandwidth of the source wavelet, it can maximise the accuracy of a finite difference operator of given order. We have shown two examples that demonstrate that this method is a significant improvement over SFD methods, and is superior also to Zhang’s OFD method in certain circumstances. This approach to calculating finite difference coefficients can be easily applied to any order of derivative, including fractional orders, and used with irregularly spaced stencils. It is also possible to extend this technique to elastic media and to consider time-space dispersion.

## Additional Information

**How to cite this article**: Yao, G. *et al*. Adaptive finite difference for seismic wavefield modelling in acoustic media. *Sci. Rep.* **6**, 30302; doi: 10.1038/srep30302 (2016).

## Figures and Tables

**Table 1:** The coefficients of finite difference for example 1.

Method	Coefficients of finite difference						
	c(0)	c(1)	c(2)	c(3)	c(4)	c(5)	c(6)
SFD	−2.98277778	1.71428571	−0.26785714	0.05291005	−0.00892857	0.00103896	−0.00006013
Zhang’s OFD	−3.12108522	1.83730507	−0.35408741	0.09988277	−0.02817135	0.00653900	−0.00092547
AFD	−3.11194944	1.82888126	−0.34750265	0.09559446	−0.02594817	0.00568842	−0.00073863

Note that only c(0) to c(6) are shown because c(−i) = c(i).

**Table 2:** The coefficients of adaptive finite difference for example 2.

Velocity(m/s)	Coefficients of adaptive finite difference						
	c(0)	c(1)	c(2)	c(3)	c(4)	c(5)	c(6)
1500	−3.14027977	1.85480309	−0.36721504	0.10776075	−0.03174054	0.00760250	−0.00107088
1600	−3.12468100	1.84038305	−0.35590670	0.10041162	−0.02797630	0.00623104	−0.00080215
1700	−3.11107063	1.82789338	−0.34632555	0.09441188	−0.02506017	0.00523960	−0.00062373
1800	−3.09916759	1.81704044	−0.33815917	0.08946136	−0.02276183	0.00450384	−0.00050080
1900	−3.08873391	1.80758047	−0.33116040	0.08533747	−0.02092257	0.00394515	−0.00041330
2000	−3.07956100	1.79930496	−0.32512820	0.08187064	−0.01942982	0.00351206	−0.00034921
2100	−3.07147026	1.79203749	−0.31989959	0.07893087	−0.01820260	0.00317008	−0.00030105
2200	−3.06430912	1.78562963	−0.31534258	0.07641786	−0.01718182	0.00289556	−0.00026405
2300	−3.05794859	1.77995741	−0.31134990	0.07425356	−0.01632371	0.00267192	−0.00023506
2400	−3.05227900	1.77491689	−0.30783409	0.07237665	−0.01559539	0.00248733	−0.00021193
2500	−3.04720807	1.77042091	−0.30472350	0.07073855	−0.01497182	0.00233318	−0.00019321
2600	−3.04265761	1.76639593	−0.30195910	0.06930043	−0.01443371	0.00220307	−0.00017783
2700	−3.03856087	1.76278019	−0.29949203	0.06803100	−0.01396598	0.00209223	−0.00016504
2800	−3.03486109	1.75952148	−0.29728159	0.06690481	−0.01355677	0.00199698	−0.00015430
2900	−3.03151011	1.75657499	−0.29529375	0.06590103	−0.01319659	0.00191449	−0.00014517
3000	−3.02846646	1.75390315	−0.29349977	0.06500247	−0.01287783	0.00184255	−0.00013736
3100	−3.02569413	1.75147319	−0.29187545	0.06419487	−0.01259428	0.00177942	−0.00013062
3200	−3.02316284	1.74925733	−0.29040018	0.06346629	−0.01234088	0.00172368	−0.00012475
3300	−3.02084565	1.74723136	−0.28905636	0.06280669	−0.01211344	0.00167420	−0.00011962
3400	−3.01871967	1.74537468	−0.28782889	0.06220758	−0.01190849	0.00163007	−0.00011509
3500	−3.01676464	1.74366903	−0.28670478	0.06166175	−0.01172312	0.00159053	−0.00011108
3600	−3.01496291	1.74209869	−0.28567278	0.06116303	−0.01155487	0.00155495	−0.00010752
3700	−3.01329923	1.74064982	−0.28472313	0.06070611	−0.01140167	0.00152280	−0.00010432
3800	−3.01175976	1.73931038	−0.28384730	0.06028642	−0.01126175	0.00149366	−0.00010146
3900	−3.01033282	1.73806965	−0.28303790	0.05990000	−0.01113360	0.00146715	−0.00009887
4000	−3.00900769	1.73691833	−0.28228834	0.05954341	−0.01101592	0.00144296	−0.00009653
4100	−3.00777507	1.73584795	−0.28159288	0.05921363	−0.01090758	0.00142082	−0.00009440
4200	−3.00662661	1.73485136	−0.28094649	0.05890803	−0.01080761	0.00140050	−0.00009246
4300	−3.00555491	1.73392189	−0.28034464	0.05862428	−0.01071515	0.00138180	−0.00009068
4400	−3.00455332	1.73305357	−0.27978331	0.05836034	−0.01062946	0.00136456	−0.00008905
4500	−3.00361586	1.73224139	−0.27925897	0.05811441	−0.01054989	0.00134861	−0.00008756
4600	−3.0027373	1.7314805	−0.2787685	0.0578849	−0.0104759	0.0013338	−0.0000862
4700	−3.0019126	1.7307667	−0.2783089	0.0576703	−0.0104069	0.0013201	−0.0000849

Note that only c(0) to c(6) are shown because c(−i) = c(i).
